# Influence of bone parameters on peri-implant bone strain distribution in the posterior mandible

**DOI:** 10.4317/medoral.19878

**Published:** 2014-08-17

**Authors:** Tsutomu Sugiura, Kazuhiko Yamamoto, Masayoshi Kawakami, Satoshi Horita, Kazuhiro Murakami, Tadaaki Kirita

**Affiliations:** 1 DDS, PhD, Clinical Instructor, Department of Oral and Maxillofacial Surgery, Nara Medical University, Nara, Japan; 2 DDS, PhD, Associate Professor, Department of Oral and Maxillofacial Surgery, Nara Medical University, Nara, Japan; 3 DDS, PhD, Assistant Professor, Department of Oral and Maxillofacial Surgery, Nara Medical University, Nara, Japan; 4 DDS, Research associate, Department of Oral and Maxillofacial Surgery, Nara Medical University, Nara, Japan; 5 DDS, DMSc, Professor and Chair, Department of Oral and Maxillofacial Surgery, Nara Medical University, Nara, Japan

## Abstract

Objectives: The success rate of dental implants depends on the type of bone at the implant site. The purpose of the present study was to investigate the effects of the bone parameters at the implant-placement site on peri-implant bone strain distributions. Study Design: The morphologies and bone densities of seventy-five potential implant sites in the posterior mandible were measured using computed tomography (CT). Based on the CT data, we defined bone parameters (low and high in terms of cancellous-bone density and crestal-cortical bone density, and thin and thick in terms of crestal-cortical bone thickness), and we constructed finite-element models simulating the various bone types. A buccolingual oblique load of 200 N was applied to the top of the abutment. The von Mises equivalent (EQV) strains in the crestal-cortical bone and in the cancellous bone around the implant were calculated. Results: Cancellous-bone density greatly affected the maximum EQV strain regardless of the density and thickness of the crestal cortical-bone. The maximum EQV strains in the crestal cortical-bone and the cancellous bone in the low-density cancellous-bone models (of 150 Hounsfield units (HU) were 1.56 to 2.62-fold and 3.49 to 5.31-fold higher than those in the high-density cancellous-bone models (of 850 HU), respectively. The crestal cortical-bone density affected the maximum EQV strains in the crestal cortical-bone and in the cancellous bone in the low-density cancellous-bone models. The crestal cortical-bone thickness affected the maximum EQV strains in the cancellous bone and in the crestal cortical-bone in the low-density cancellous-bone models. Conclusions: Our results confirm the importance of bone types for the peri-implant bone strain distribution. Cancellous-bone density may be a critical factor for peri-implant bone strain.

** Key words:**Dental implant, bone density, finite-element analysis.

## Introduction

Osseointegrated dental implants are widely used for functional and aesthetic rehabilitation. Although high initial success rates of implants have been reported, there are numerous failures in areas where bone density is low (1). Excessive stress/strain at the bone-implant interface can cause peri-implant bone defects and osseointegration failure (2). A key factor for the success of a dental implant is the manner in which the stresses/strains are transmitted to the surrounding bone (3). Finite-element analysis (FEA) has been widely used to evaluate the effect of bone type on peri-implant stress/strain because FEA allows researchers to predict stress/strain distribution in the bone in contact with the implants. Many authors have investigated the effects of bone parameters, such as crestal cortical-bone thickness, and density of cortical bone and cancellous bone on peri-implant stress/strain distributions. The studies have demonstrated that a greater cortical-shell thickness and a higher cortical and cancellous-bone density reduce the stress/strain concentrations around the implants (4-10). However, the importance of each bone parameter remains unclear for clinical situations because various values of the bone parameters were assumed in the studies (4-11). Therefore, biomechanical analysis using bone parameters based on patients’ data is necessary to provide clinicians precise understanding of the relationship between bone type and the peri-implant stress/strain distribution during preoperative planning.

In the present study, we measured bone morphology and density of the mandible in preoperative patients using computed tomography (CT). Next, bone parameters were defined. Based on these data, three-dimensional (3D) finite-element models simulating different bone types were prepared. The purpose of this study was to investigate the influence of bone parameters at the implant-placement site on peri-implant bone strain distribution.

## Material and Methods

-Radiological evaluation of the bone

CT images were obtained from 34 patients who were to be implanted with fixed prostheses [18 females aged 59 ± 7 years (mean ± S.D.), ranging 40 to 68 years; and 16 males aged 54 ± 11 years, ranging 41 to 70 years]. Seventy-five potential implant sites were identified at the posterior region of the mandible (4 mm to 20 mm posterior to the mental foramen). The CT scans were performed using a spiral CT machine (LightSpeed Ultra16, General Electric, Milwaukee, WI, USA) with the following technical parameters: tube voltage 120 kV, tube current automatic current modulation, slice thickness 0.625 mm and slice intervals 0.625 mm. The patients were either fully or partially edentate and had potential implant-placement sites with lengths greater than 8 mm and diameters greater than 4.1 mm. Patients with an incompletely healed socket (because of recent tooth loss) were excluded. All experimental procedures were conducted with the ethical approval of the Nara Medical University.

-Measurements of bone morphology and bone density

**Figure 1:**
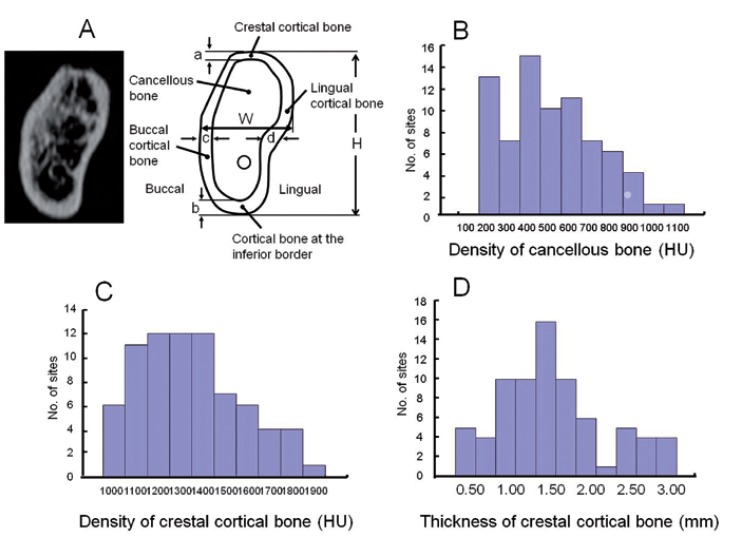
A) Image and measurements of implant-placement sites in the posterior region of the mandible, 4 mm to 20 mm posterior to the mental foramen. The height (H), width (W), and thickness of the buccal, lingual, crestal and inferior cortical bone of the mandible were measured. The mean densities of the cancellous bone and the crestal cortical-bone of the implant area, and the buccal, lingual, and inferior cortical bone were measured. Cortical thickness; a = alveolar crest, b =inferior border, c = buccal, d = lingual. B) Distribution of the density of cancellous bone, C) Distribution of the density of crestal cortical-bone, D) Distribution of thickness of the crestal cortical-bone.

The height, width, and thickness of the crestal, inferior, and lateral cortical bones were measured in the posterior region of the mandible, 4 to 20 mm posterior to the mental foramen. The mean density of the cancellous bone and the crestal cortical-bone of the implant area, and the buccal, lingual, and inferior cortical bones were measured using medical imaging software (SimPlant, Materialise, Leuven, Belgium) (Fig. 1). Bone morphologies and bone densities are summarized in tables  Table 1 and  Table 2. The distributions of the bone densities (measured in Hounsfield units (HU)) and the crestal cortical-bone thicknesses are shown in figure 1.

**Table 1:**
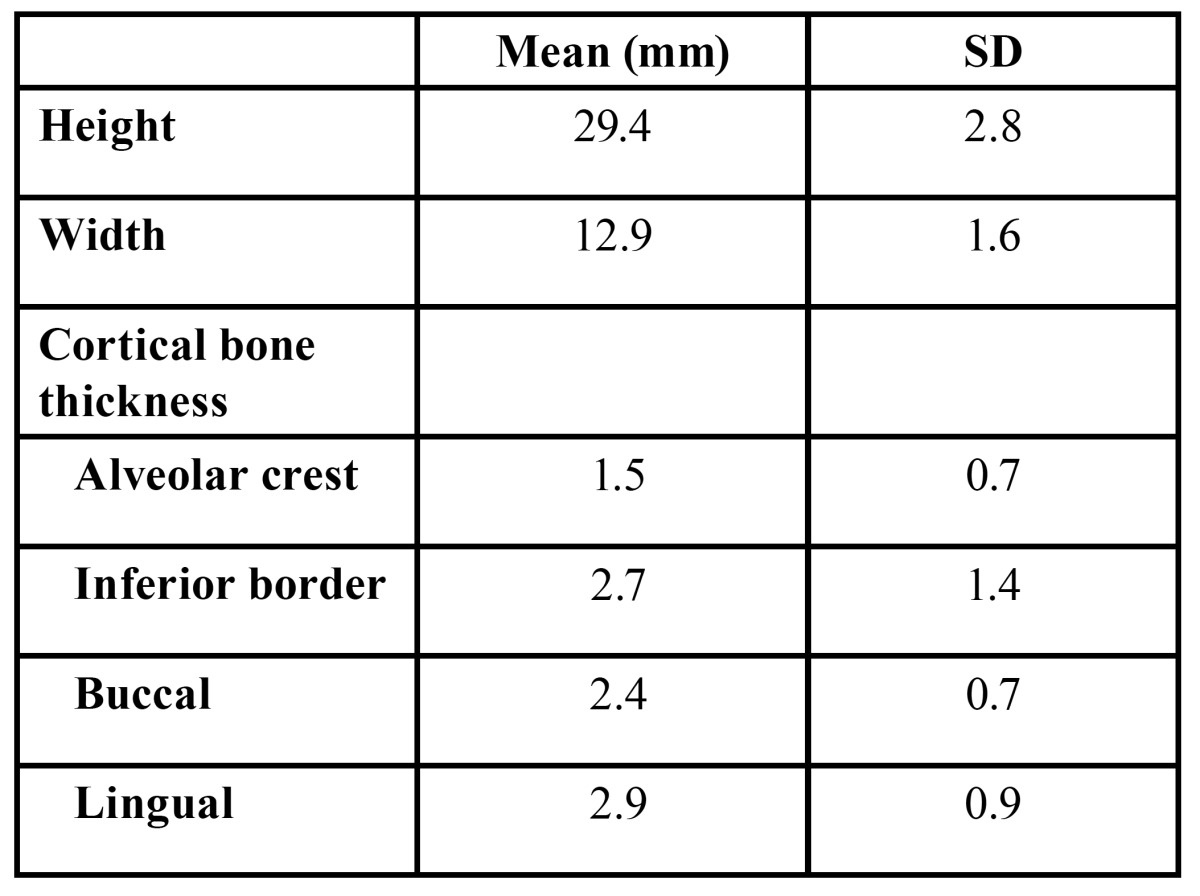
Morphological measurements.

**Table 2:**
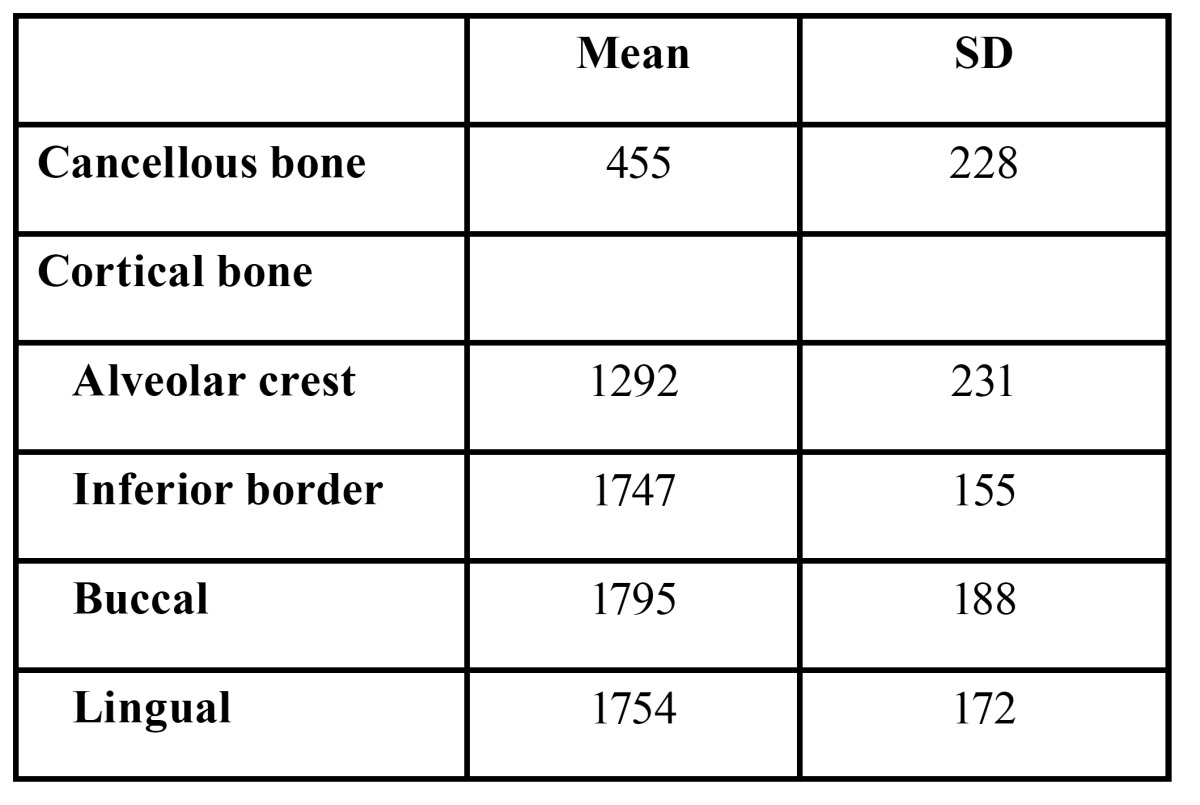
Bone density (HU).

-Definition of bone parameters and material properties

We defined three independent bone parameters, including thickness and density of the crestal cortical-bone and cancellous-bone density at the implant site. The Kolmogorov-Smirnov test showed that all of the parameters were normally statistically distributed. We defined the 5th and 95th percentiles of bone density (950 HU and 1750 HU for the crestal cortical-bone, 150 HU and 850 HU for the cancellous bone) as low and high, respectively. Similarly, the 5th and 95th percentiles of the crestal cortical-bone thickness (0.4 mm and 2.8 mm) were defined as thin and thick, respectively. Because the cortical bone density of the buccal, lingual and inferior border of the mandible showed similar values, a mean value of 1765 HU was defined as the bone density of these areas. A linear regression equation was created based on the CT values of the calibration phantom. Using these calibrated CT data, each bone density measured in HU was converted to a bone mineral density expressed in g/cm3 ( Table 3).

**Table 3:**
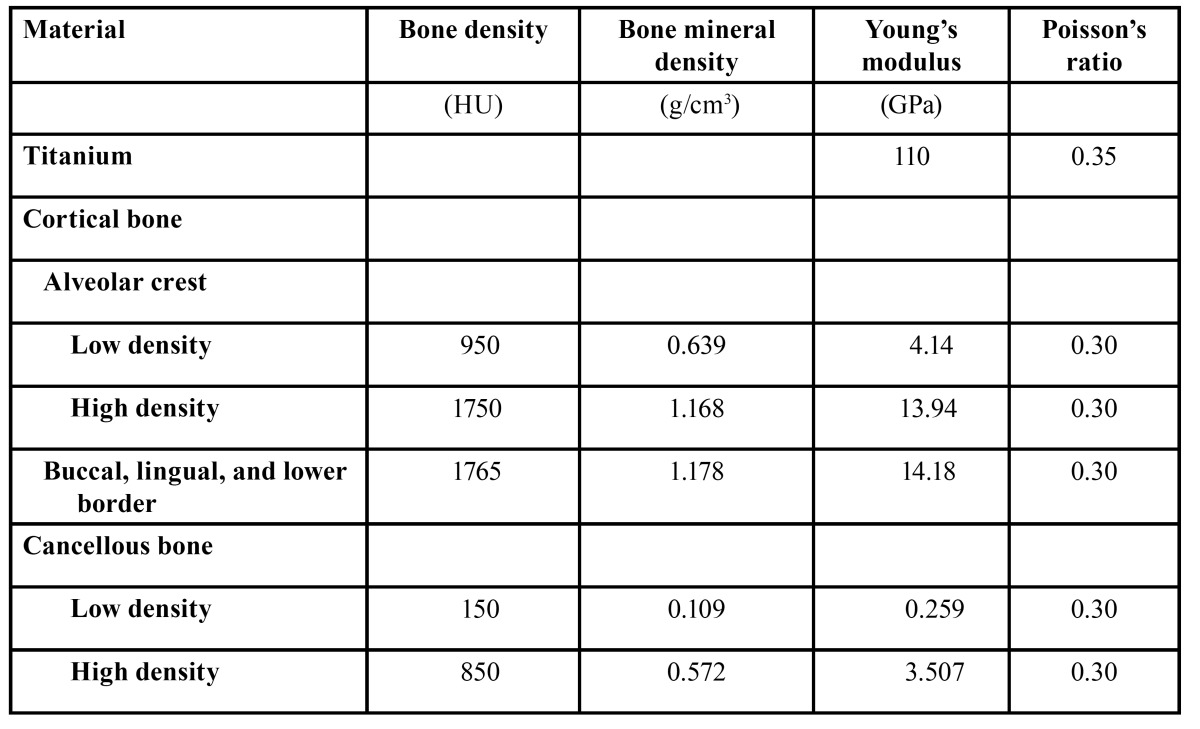
Table 3. Bone density and material properties.

-Finite-element models

**Figure 2:**
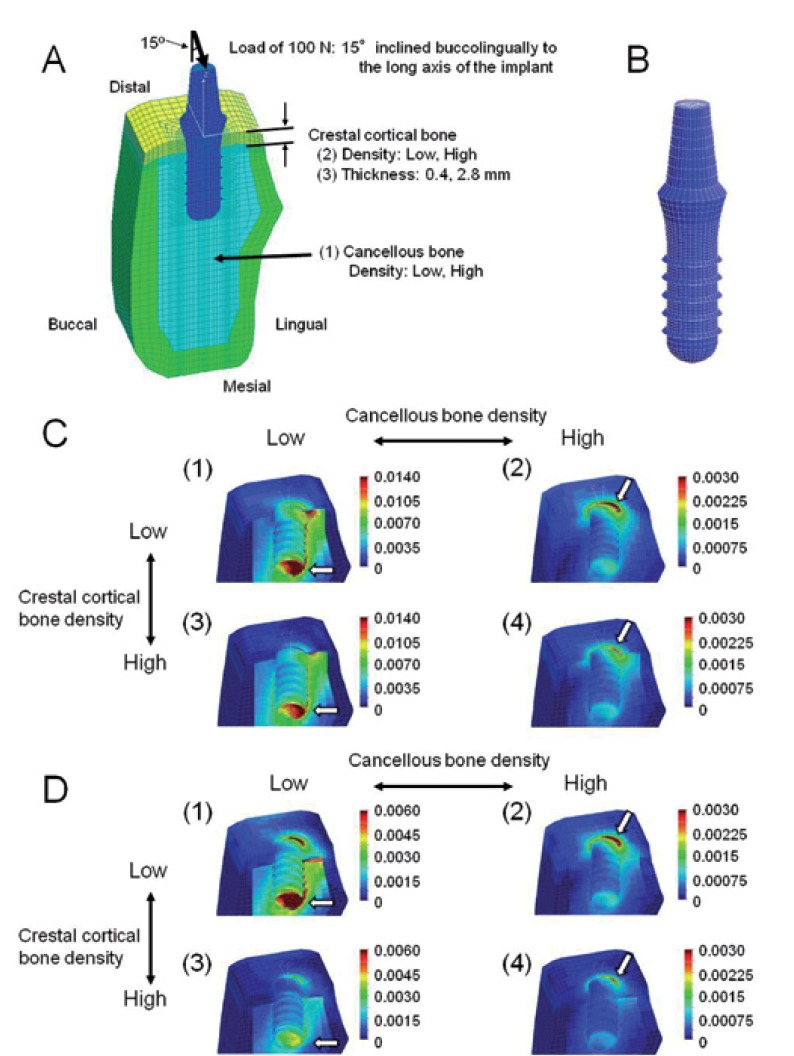
A, B) Finite-element model. A) Cross-sectional view of the symmetry plane. The three independent parameters include: (1) density of cancellous bone, (2) density of crestal cortical bone, (3) thickness of crestal cortical bone. B) Implant and abutment. C) Equivalent strain distribution in models with thin (0.4 mm) crestal cortical-bone. The implant is removed in this illustration. (1) Model with low-density cancellous and crestal cortical-bone, (2) model with high-density cancellous-bone and low-density crestal cortical-bone, (3) model with low-density cancellous bone and high-density crestal cortical-bone, (4) model with high-density cancellous and crestal cortical-bone. The arrows indicate sites where the peak EQV strains were generated. D) Equivalent strain distribution in models with thick (2.8 mm) crestal cortical bone. The implant is removed in this illustration. (1) Model with low-density cancellous and crestal cortical-bone, (2) model with high-density cancellous bone and low-density crestal cortical-bone, (3) model with low-density cancellous bone and high-density crestal cortical-bone, (4) model with high-density cancellous and crestal cortical-bone.

A cross-sectional view of an edentulous mandible in the right second premolar region was digitized and used as a base for the mandibular model using average values of the CT data ( Table 1). A 3D model was created using FEA software (COSMOS/M, Structural Research & Analysis Corporation, Los Angeles, CA, USA). A Straumann threaded implant (Institut Straumann, Waldenburg, Switzerland) with a 4.1-mm diameter and a 10-mm length was simulated in this study. An implant and a 6-mm abutment were modeled as one piece (Fig. 2). This implant was assumed to be completely osseointegrated at the implant/bone interface. The material properties were assumed to be homogeneous, isotropic, and linearly elastic. The Young’s modulus for each bone mineral density was calculated using the equations proposed by Keyak (12) ( Table 4). The Poisson’s ratios of the bones and the material properties of the implant were obtained from previous data (6) ( Table 3). The finite-element model consists of eight-node hexahedral elements, with approximately 21500 elements and 22400 nodes.

**Table 4:**
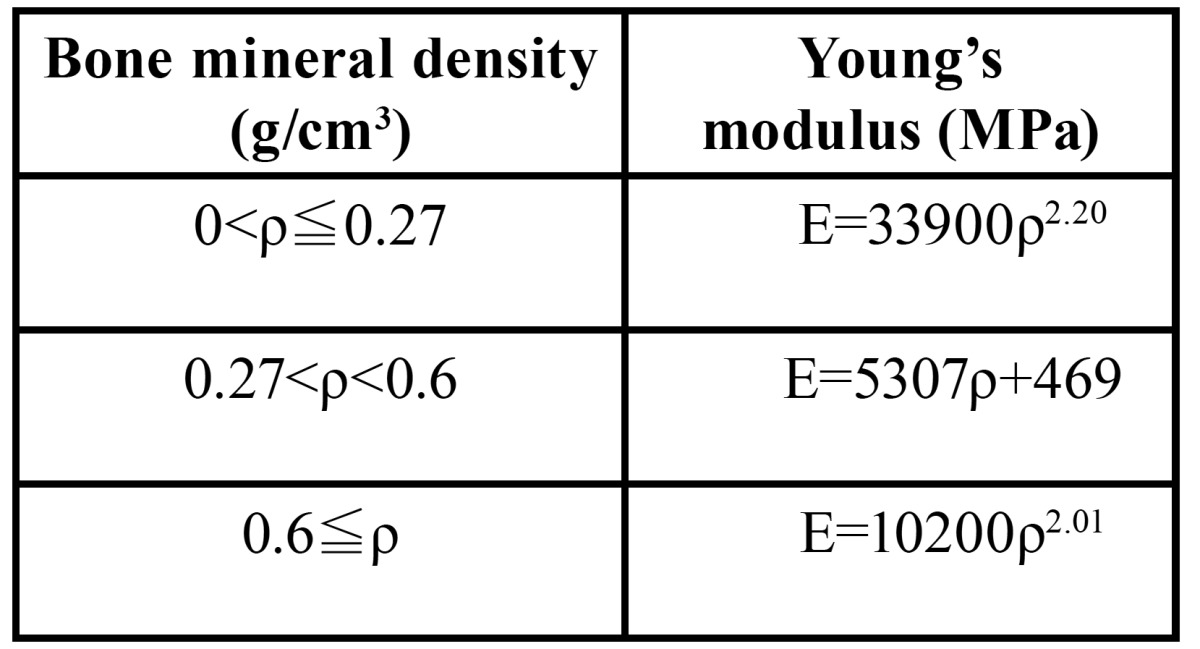
Relationship between bone mineral density and Young’s modulus.

-Loads and constraints

Based on the data of the mean occlusal force recorded in patients with implants (13,14), a buccolingual oblique load of 200 N was applied to the top of the abutment. Based on previous FEAs (14,15), the oblique loading angle was defined as 15 degrees to the axis of the implant. As a symmetric half model of the mandible was used, only half of these loads were applied. For boundary conditions, the nodes of the distal end of the model were displaced in all directions. Due to the symmetry of the model, symmetric boundary-conditions were prescribed at the nodes that were on the plane of symmetry (mesial end) (Fig. 2).

-Analysis of bone strain

The von Mises equivalent (EQV) strains of the bone were calculated because EQV stresses/strains are commonly reported in FEA studies to summarize the overall stress/strain state at a point (5,6).

## Results

-Strain distributions in bone around the implant

**Figure 3:**
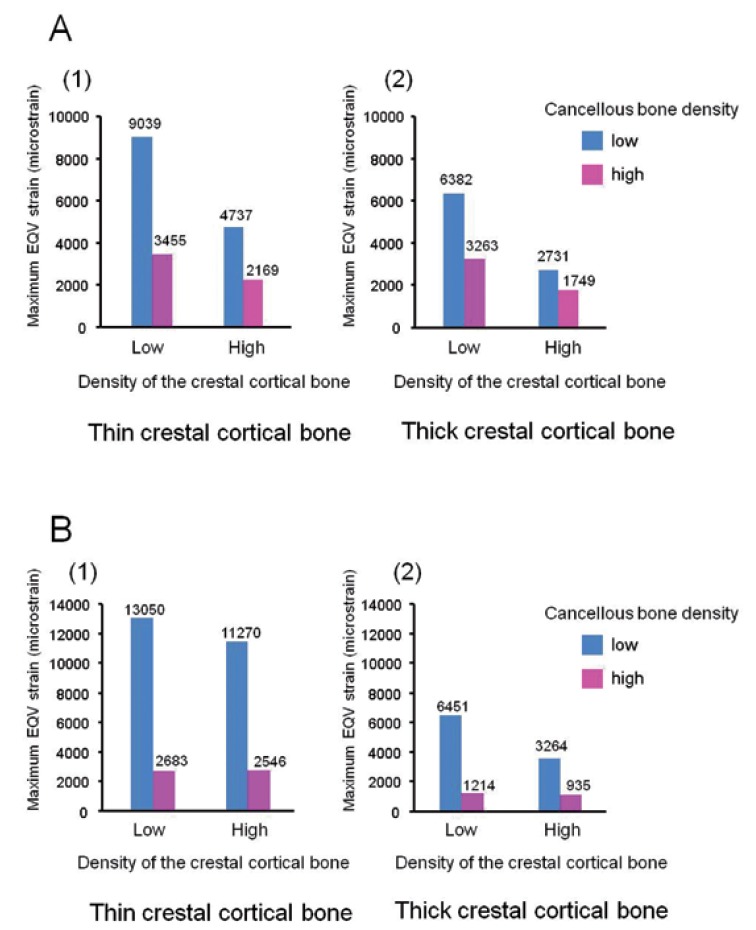
A) Maximum equivalent strain in the cortical bone. B) Maximum equivalent strain in the cancellous bone.

The EQV strains were generally high in the crestal cortical-bone around the implant, and in the cancellous bone around the neck, the tip of the thread and the lingual apex of the implant. In the low cancellous-bone density models, the strains were concentrated in the bone around the neck and the apex of the implant (Figs. 2,3). In contrast, the strains were concentrated in the bone around the neck of the implant in the high cancellous-bone density models (Figs. 2:4).

-Cortical bone strain

The highest maximum EQV strain (9039 microstrain (με)) was observed in the low cancellous-bone density model with low crestal cortical-bone density and thin crestal cortical-bone (Fig. 2). The maximum EQV strains were higher in the low cancellous-bone density models than those in the high cancellous-bone density models (1.56-2.62-fold). The maximum EQV strains were also higher in the low crestal cortical-bone density models than in the high crestal cortical-bone density models (1.59-2.34-fold). The crestal cortical-bone thickness did not affect the maximum EQV strains in the high cancellous-bone density models very much (1.06-1.24-fold higher in the thin crestal cortical-bone models). However, it did slightly influence the maximum EQV strains in the low cancellous-bone density models (1.42-1.73-fold) (Fig. 3).

-Cancellous-bone strain

The maximum EQV strain was observed in the cancellous bone around the apex of the implant in the low cancellous-bone density models (arrows in Figs. 2,3), and in the cancellous bone around the neck of the implant in the high cancellous-bone density models (Figs. 2:4). The highest maximum EQV strain (13050 με) was observed in the low cancellous-bone density model with a low crestal cortical-bone density and a thin crestal cortical-bone (Fig. 2). The maximum EQV strains were much higher in the low cancellous-bone density models than in the high cancellous-bone density models (3.49-5.31-fold). The crestal cortical-bone density did not affect the maximum EQV strains in the high cancellous-bone density models very much (1.05-1.30-fold higher in the low crestal cortical-bone density models). In contrast, this density did slightly influence the maximum EQV strains in the low cancellous-bone density models (1.16-1.98-fold). The maximum EQV strains were higher in the thin crestal cortical-bone models than in the thick crestal cortical-bone models (2.02-3.45-fold) (Fig. 3).

-Risk for bone fatigue failure

A microstrain level that is over 4000 is commonly indexed as the threshold for bone-fatigue failure (16-18). The maximum EQV strains were under 4000 με in the crestal cortical-bone and the cancellous bone in the high cancellous-bone density models (for any crestal cortical-bone density and thickness). In contrast, the maximum EQV strains were over 4000 με in the low cancellous-bone density models. This finding was observed in most cases, except for the models with high crestal cortical-bone density and thick crestal cortical-bone (Fig. 3).

## Discussion

Excessive strain can cause damage to the implant-bone interface and to the microstructure of the bone and thus cause a loss of osseointegration with the implant (2). Therefore, the physiological limits of strain in the peri-implant bone should be taken into account before the placement of a dental implant. This study investigated the influences of bone parameters on the peri-implant strain distributions in the posterior mandible. There are a lot of FEAs in which the effects of bone parameters on peri-implant stress/strain distributions have been studied. The validity of the simulations depends on morphology, material properties, boundary conditions, and the bone-implant interface (3). The bone morphologies and Young’s moduli of cortical bone and cancellous bone in human mandibles vary greatly across individuals and sites (7). In previous FEAs, Young’s modulus was assumed to be from 0.231 to 1.10 GPa for low-density cancellous bone and from 1.37 to 9.5 GPa for high-density cancellous bone (4-7,11,19). In the present study, we defined the morphologies and the values of Young’s modulus of the mandible based on the CT data of the patients to improve the validity of the finite-element model.

It has been reported that the mean bone density ranged from 306 to 721 HU at implant placement sites in the posterior mandible (20-23). Our data revealed that the mean bone density of cancellous bone was 455 ± 228 HU. This is consistent with previous data that indicated a cancellous-bone density of 360 HU in the posterior mandible (23). The cortical bone density of the mandible ranges from 1000 to 1800 HU (24,25). All of the mean values of the cortical bone density in our study were within this range.

De Oliveria *et al*. (20) used criteria based only on the cancellous-bone density, in which more than 400 HU is categorized as type 1 bone, and less than 200 HU as type 4 bone in the classification by Lekholm and Zarb (20). Norton and Gamble (25) categorized more than 850 HU as type 1, 500-850 HU as type 2 or 3, and 0-500 HU as type 4. Therefore, high-density cancellous bone (of 850 HU) and low-density cancellous bone (of 150 HU) in this study would be equivalent to type 1 or 2 bone and type 4 bone, respectively.

In the low-density cancellous-bone models, the maximum EQV strains were observed in the cancellous bone around the implant, whereas in the high-density cancellous-bone models, the maximum strains were generated in the bone around the cervical area of the implant. The stress/strain is generally concentrated in the cortical bone around an implant neck (4,5). However, if the cancellous bone is soft, the cancellous bone does not resist the load efficiently and a larger strain is generated. Our data are in agreement with the results reported by Tada *et al*. (5), which indicated that the maximum EQV strain values in cancellous bone were approximately 3500 με and 9000 με in type 1 bone models and in type 4 bone models, respectively, under loading conditions of 200 N.

The maximum EQV strain values in alveolar bone greatly depended upon the cancellous-bone density around the implant. These results are consistent with those reported by Guan *et al*. (8), who evaluated the influence of bone parameters on the peri-implant stress distribution. An increase in cancellous-bone density can relieve the peri-implant stress/strain concentration because it provides a greater bone-implant contact surface. Therefore, the higher the cancellous-bone density, the lower is the peri-implant strain that develops in the alveolar bone.

The crestal cortical-bone thickness is also believed to be an important factor for the success of implants (26). Cortical and cancellous bone stresses decrease with an increase in the crestal cortical-bone thickness (6-8,26). The present study also showed that crestal cortical-bone thickness had a significant effect on the cancellous-bone strain. In the thick crestal cortical-bone models, strains in the cancellous bone were reduced to less than half, even with low cancellous-bone density. A thin crestal cortical-bone is easily deformed by occlusal overload. The loads are further transmitted to the cancellous bone, and thus increase EQV strains in the cancellous bone.

Occlusal overloading may cause pathological stress/strain and stimulate bone resorption (2). A micro strain level that is over 4000 is commonly indexed as the threshold for bone fatigue micro fracture (16-18). Our data indicated that the maximum EQV strains in high-density cancellous-bone models were under 4000 με (in any of the crestal cortical-bone models). The EQV strains in the low-density cancellous-bone models were over 4000 με even in the high density or thick crestal cortical-bone models. These results suggest that cancellous-bone density is a critical factor for the peri-implant strain.

There were a number of limitations in this finite-element model: the simplified shape of the mandible, its homogenous and isotropic structure and linear elasticity. In our FEA, only crestal cortical-bone thickness was changed because the crestal cortical-bone thickness seems to be important for peri-implant bone stress/strain distributions (rather than the buccal, lingual and inferior cortical-bone thicknesses) (9,10,27). The bone-implant and implant-abutment interfaces were also assumed to be completely bonded. A load of 200 N was applied in a fixed direction. The applied loading was static, although bone responds to dynamic loads, rather than to static loads (28). Because the models did not accurately reproduce the complex forces that are exerted during chewing, the strains that were obtained were reference values, and they cannot be directly compared with the threshold strain of 4000. These limitations should be taken into account when applying our results to a clinical situation.

## Conclusions

Within the limitations of this study, cancellous-bone density may be a critical factor for peri-implant bone strain. The maximum EQV strains around the implant in the crestal cortical-bone and the cancellous bone in the low-density cancellous-bone models (of 150 HU) could be 2.62-fold and 5.31-fold higher than those in the high-density cancellous-bone models (of 850 HU), respectively. The maximum EQV strain was low in the high-density cancellous-bone models, regardless of the density and thickness of the crestal cortical-bone.
